# Medical students’ understanding of clinical empathy – a qualitative exploratory interview study

**DOI:** 10.1186/s12909-024-06428-5

**Published:** 2024-12-05

**Authors:** Nadine Janis Pohontsch, Anne Stark, Martin Scherer

**Affiliations:** Department of General Practice and Primary Care, University Medical Center Hamburg-Eppendorf, Martinistr. 52, 20246 Hamburg, Germany

**Keywords:** Medical education, Empathy, Qualitative research

## Abstract

**Background:**

Empathy plays an important role in the interaction and communication with patients. Physicians’ empathy has various positive patients’ and physicians’ outcomes. Despite the inclusion of empathy in medical curricula and the relevance of empathy in general and physicians’ concept of it to medical care, there is no common definition of empathy in the clinical context: definitions tend to be abstract and we do not know enough about medical students’ conceptualization of clinical empathy. A clear and consensual definition of empathy is needed to be able to teach and measure empathy adequately. We aimed to explore German medical students’ views and understanding of (clinical) empathy.

**Methods:**

We interviewed 24 students from the second half of the 3rd year and in their final clinical year (six female and male students in each subgroup) using a semi-structured interview guide. Interviews were digitally recorded and transcribed verbatim. We analysed the transcripts using thematic synthesis (Braun & Clarke).

**Results:**

We found three overarching themes: (1) empathy means perceiving and understanding patients’ needs and acting accordingly, (2) empathy as an interpersonal, intangible construct and (3) taking time for patients. Showing interests, impartiality and openness towards the patients as well as the need to take patients seriously, treating them with respect, having a holistic view on patients and generate some kind of closeness with patients are subthemes of the first overarching theme.

**Conclusions:**

Although it is often stated that the various existing definitions of empathy are abstract or far from practice, German medical students seem to have a good idea how to define empathy. Their definition resembles definitions known from the literature and used in education. Further research is needed to compare concepts of empathy of medical students from different countries and cultural backgrounds to inform research and teaching. It would also be interesting to investigate how concepts of empathy change over the course of study and affect perceptions of empathy in third party assessments.

## Background

Empathy plays an important role in the interaction and communication with patients. Physicians’ empathy has various positive patients’ outcomes (e.g., increased patient satisfaction, participation and compliance, reduced distress and anxiety or better psychosocial adjustment [1–4]). Empathy is also beneficial for physicians since it may lead inter alia to better diagnostics, more job satisfaction and less burnout [2, 4–6]. Increasing medical students’ empathy might therefore lead to more positive outcomes on both sides and much research focuses on factors de- or increasing empathy during the education of medical students (e.g [7, 8]).

Jeffrey [9] points out that a doctor’s definition of empathy might affect how they think about professionalism and approach patients. There is no common definition of empathy in the clinical context [10–12]. Jeffrey [11], for example, offers a broad concept of clinical empathy containing elements of connection, clinical curiosity, another-oriented perspective, self-other differentiation and care. Different authors come to different, partially overlapping definitions. Morse et al. [13] identified in their review four components of clinical empathy (emotive, moral, cognitive and behavioural). Similar theoretical considerations were described by Neumann et al. [5] in an overview of the “nature” of clinical empathy. However, these components are not included in every concept or definition.

Literature on the concept of clinical empathy highlights again and again a tension between those who see empathy as a more intellectual (cognitive) ability and those who argue that empathy also includes an emotional component [4, 9, 10, 14–16]. A commonly used definition of clinical empathy [17, 18] is that of Mercer and Reynolds [19]. According to Mercer and Reynolds, clinical empathy includes the “ability to understand the patient’s situation, perspective and feelings (and their attached meanings); to communicate that understanding and check its accuracy; and, to act on that understanding with the patient in a helpful (therapeutic) way” [ [19], p. 9].

Hall et al. [12] showed significant differences between what (non-medical) students, community members, oncology patients and physicians considered to be clinical empathy. Relationship orientation was more dominant in cancer patients and physicians than in other groups, while emotional involvement was more dominant in physicians than all other groups. According to another study from Hall et al. [20] laypeople also differed widely in their conceptualization of empathy. Schwartz et al. [21] found clinicians’ concept of empathy to extend over the affective component of empathy to structural and organizational factors conveying or reinforcing empathy, e.g. making time for patients. Clinicians’, medical students’, patients’, laypersons’, researchers’ and philosophers’ view of empathy may differ a lot from each other and may also differ from a definition that matters in practice [13, 16, 20–22]. Different conceptualizations of clinical empathy have influences on teaching, medical care and the correlation of patients’ perception on clinicians’ empathy and clinicians’ self-judgement concerning clinical empathy [9, 12].

Medical students’ development of empathy in the context of medical education and care is given much attention, since they are the doctors of tomorrow (e.g [14, 23]). Developing and strengthening empathy in medical students has become more central in many medical curricula, but the teaching of empathy is diverse and outcomes are unclear [24, 25]. At the same time, many studies investigating students’ perspectives on empathy focus on aspects like barriers and facilitators of empathy and offer only small or shallow insights into the students own concept of empathy [16, 26–28].

The medical curriculum of the University of Hamburg allows the students to acquire necessary practical skills and abilities as well as psycho-social skills for the medical profession. Psychosocial skills were conveyed by teaching theoretical basics and social interactions with (simulated) patients and colleagues. Psychosocial skills comprise psychological, social-scientific and basics of behavioural medicine with a special focus on doctor-patient relationship and basics of patient-oriented medicine. Students report hands-on-experience, role models, and emphasis on the importance of empathy in the curriculum to foster empathy development during their course of studies [7].

However, despite its inclusion in medical curricula, the relevance of empathy in general and physicians’ concept of it to medical care, we do not know enough about medical students’ conceptualization of clinical empathy. Jeffrey [16] conducted a meta-ethnography of eight interview-based qualitative research studies on medical students` views and experiences of empathy and found strongly varying concepts of empathy. A more recent systematic review by Costa-Drolon et al. [24] dealt with 35 articles on the topic of medical students’ perspectives on empathy and, unsurprisingly, repeated the finding of medical students’ confusion in defining empathy. Despite the great amount of literature reviewed, the authors did not find a study from Germany approaching the question how medical students define or conceptualize empathy [24]. It cannot be ruled out that the definitions and expressions of empathy vary among medical students in different countries due to different medical curricula and cultural differences (e.g [29–31].

In conclusion, we still do not have a unified definition of (clinical) empathy and the question how to best teach empathy and how to increase empathy in clinical practice remains unsolved [24]. A clear and consensual definition of empathy is needed to be able to teach and measure empathy adequately. To be able to get a holistic view on (possible) definitions and important aspects of (clinical) empathy studies from different countries and different medical groups (e.g. students, general practitioners and physicians from other specialties) are needed.

We conducted a comprehensive qualitative study with German medical students in different stages of their education with the aim to explore their views and understanding of (clinical) empathy.

## Methods

We conducted a qualitative interview study with medical students in the second half of the 3rd year and students in their final clinical year (6th year of medical studies). We aimed to gain an in-depth understanding of their views on (clinical) empathy. All students studied at the University Medical Center Hamburg-Eppendorf in Germany where the study center was located. Our research was funded by the Research Promotion Fund of the Faculty of Medicine (NWF 16/04). Further details and results of the study are published elsewhere [7].

### Participants

We choose a purposive sampling approach [32] to select interviewees. Female students usually have higher scores on empathy measures than male students [2, 33]. Studies show that there might be a decline in students’ empathy during their medical education and one can expect that hands-on experiences with patients in the clinical environment influence students’ empathy [34, 35]. Based on these findings we choose to interview female and male students from the second half of the 3rd year and those in their final clinical year to maximize variations in the students’ accounts on their understanding of empathy.

### Recruitment

A staff member of the deanery invited the eligible students to take part in the study via email. To be eligible students had to be in the second half of the 3rd year or in their final year doing their clinical internship (inclusion criteria). The only exclusion criterion was insufficient knowledge of the German language.

Students willing to participate contacted the study team by email or phone, received written and oral information about the study, where able to ask further questions and provided written, informed consent before the interviews were conducted. Participation in the study was voluntary and non-participation had no negative consequences. Participants received an allowance of 25€.

### Interview guideline and interview conduction

The interview guideline was developed by NJP and AS aiming at producing elaborate accounts of students’ understanding of and experiences with empathy. The interview guideline (for full guideline see also [7]) included the following topics: short introduction of interviewer and study, subjective definition and meaning of empathy in the clinical setting, handling patients’ emotions, reasons for (not) being empathic towards patients, differences between personal and clinical empathy, factors promoting and hindering empathy, ideas for teaching empathy and self-reflection concerning the student’s empathy during the course of studies.

AS, who was unknown to all study participants before the study, conducted all semi-structured qualitative interviews [36, 37] at the Department of General Practice and Primary Care of the Medical University Center Hamburg-Eppendorf in spring 2016. All interviews took place in quiet rooms of the department and only the interviewee and AS were present. The semi-structured guideline incorporating open-ended questions allowed interviewees to talk openly about their experiences while allowing the interviewer to guide interviewees concerning subjects of interest. All interviews were digitally recorded and transcribed verbatim by a trained research assistant following designated transcription rules (including rules for anonymization of the material).

### Data analysis

We analysed the interview data using Braun & Clarke’s thematic analysis [38, 39], a method for identifying, analysing, and reporting themes within qualitative data. It combines minimal organization with a rich detailed description of the dataset [38]. We chose the semantic approach [38] focusing on the identification of explicit meanings of the data based on what was overtly stated by the interviewees. We therefore followed a realist paradigm [40], meaning that we assume that perception of reality is influenced by our perspective of it.

Thematic analysis includes the following steps: familiarizing yourself with your data, generating initial codes, searching for themes, reviewing themes, defining and naming themes and finally producing the report [38]. While overarching themes represent central ideas in the data, subthemes represent particular aspects of that theme [41]. The data was managed using MAXQDA 11 (Verbi GmbH). AS and NJP familiarized themselves with the data by producing and discussing case summaries for every interview. AS conducted the inductive coding and theme development process in close consultation with NJP. To secure the intersubjective comprehensibility and credibility of the analysis [42], the results were presented to and discussed with MS as well as with an interdisciplinary work group for qualitative methods led by NJP.

## Results

### Participants

We interviewed 12 female and 12 male students [six being in the second half of the 3rd year and six in their 6th (final) year respectively]. Students in the second half of the 3rd year were between 21 and 29 years (mean 24 years) and students in their final year 24–43 years (mean 29 years) old with a mean number of 6,5 completed years.

Interviews lasted between 35 and 90 min (mean 57 min). Interviewee IDs (in the results section) were structured to indicate the interviewee’s number and status (Interview_01–12 for students in the second half of the 3rd year and Interview PJ_01–12 for students in their final clinical year).

### Qualitative analysis

We found three overarching themes: (1) empathy means perceiving and understanding patients’ needs and acting accordingly, (2) empathy as an interpersonal, intangible construct and (3) taking time for patients. Overarching theme 1 has six subthemes as displayed in Table 1. The hierarchy of themes displayed in Table 1 structures the following results section.

**Table 1:** Hierarchy of themes

**Overarching themes**	**1) Empathy - perceiving and understanding patients**’ **needs and acting accordingly**	
		**Subthemes:** • Being interested in patients, their life situation, and their needs • Impartiality towards patients • Taking patients’ complaints and needs seriously • Holistic view on patients • Closeness and familiarity between physician and patient • Treating patients respectfully
	**2) Empathy as an interpersonal, intangible construct**	
	**3) Taking time for patients**	

### Overarching theme 1: Empathy - perceiving and understanding patients’ needs and acting accordingly

The perception of patients’ needs and state of mind plays a central role in the medical students’ understanding of clinical empathy. This means to feel how the patient is doing, to notice if there are feelings like sadness, shame, insecurity, or fear or to recognize if there are certain unspoken needs.

*“Empathy means first of all [to get involved] in the […] emotional level of the other person / first of all to feel and […] (…) to react in consideration of this […] of course you have your expectations of how someone deals with a message*,* but […] of course I still have to feel where the patient is at the moment. Is he already processing? Can he absorb it at all right now? Or is he just taking in pure information*,* but can’t process it emotionally at the moment? […] And I think that is ultimately the difficulty or the high art of empathy*,* to first of all cautiously approach it and to feel where the other person is in order to be able to present the information in the right form.”*

(Interview PJ_09, paragraph 2)

It is also central to clinical empathy to understand patients’ needs, emotions and behaviours, to feel into and with patients, to understand their individual situation and to put oneself in the patient’s shoes.

*“I think empathy is a difficult concept to define*,* but I would say that in the doctor-patient relationship it is important to get involved with the patient and to really try to understand what moves this person*,* what he is afraid of*,* what his worries are*,* what his hopes are*,* what is on his mind*,* and then to build as good a relationship as possible with this patient and to be in the medical profession able to help him adequately.”*

(Interview_07, paragraph 2)

Another key aspect of empathy is taking into account patients’ needs and feelings and acting in an adapted way. An empathic behaviour includes to show patients verbally or non-verbally that you understand her or him and to address perceived emotions, worries or insecurities, hence patients notice that they are recognized with their concerns. However, students believed that clinical empathy does not always mean being nice and telling patients what they want to hear. An empathic behaviour may also involve to be more rigorous in order to help patients to recover or to feel better. A more rigorous approach appears right, if patients for example show health damaging behaviour or refuse vital treatment.

*Well, there are situations that if you are simply […] nice to a patients then it has no consequence whatsoever for the patient, he/she doesn’t get it at all. And I believe that there are situations in which one has to tell the patient/one is empathic, one’s trying to put him-/herself in the patient’s shoes, one knows what he/she needs in that moment and says in a more informal way ‘Buddy, that’s not on right now.’*

(Interview_PJ_06, paragraph 8)

### Subthemes

The following subthemes represent differential aspects of empathy from the interviewees’ point of view.

#### Being interested in patients, their life situation, and their needs

Empathic behaviour includes to be focussed on patients, and to take time to listen attentively and interested to patients’ narratives without interrupting and without doing other things, e.g. looking at the computer screen or beginning to document the consultation. Even if one has only little time for patients, it is possible to approach patients with interest and attention. Moreover, empathic behaviour includes doctors leaving patients room for own thoughts, questions or emotions and asking patients questions about the disease, well-being, illness-related restrictions and therapy respectively. Students believed that it is also empathic to ask further questions about personal topics like plans and hobbies or to ask questions about feelings regarding the disease. Consideration of patients’ needs like whether the window should be opened was considered empathic too.

*“I think you just have to show interest and ask. So*,* that is*,* I think the most important thing*,* that you feel somehow taken care of or understood and not somehow either misunderstood or labelled.”*

(Interview PJ_05, paragraph 8)

From the students’ point of view empathic behaviour in the clinical context means that doctors consider their patients as partners and talk with them “at eye level” (figuratively speaking, Interview_12, paragraph 32). This may include that doctors and patients confer together what further treatment should look like.

*“I think I would like a doctor who treats me to also say: “Oh yes*,* I see the lab results are good*,* but somehow you don’t feel well”*,* not someone who pulls off his plan like an apparatchick. And says: ‘Yes*,* great*,* medication is at its optimum.’*,* but the patient is vomiting all the time. Or has diarrhea*,* for example*,* or something. That also happens*,* it’s just a different image of medicine. So the other one is more at eye level with the patient*,* I would say. And (…) the other is more like: “Okay*,* I’m super well informed*,* the patient has no plan and that’s why I’m making my plan now and he has to carry out the plan the way I imagine it”.*

(Interview PJ_01, paragraph 62)

Moreover, interviewees consider it as empathic to be informed about new patients’ medical histories during medical visits (sparing patients the need to retell their medical history). They also see it as crucial to convey the feeling to patients that they are approachable for questions.

#### Impartiality towards patients

Impartiality and openness towards patients, their situation and their problems are also components of empathy according to our interviewees. Both help to understand and respond to patients in the best possible way. Interviewee_03 explained what he understood by an empathic doctor being open:

*“He has an open attitude towards me. That means he is not prejudiced […]. Well*,* there are people […] who are not financially rich or who have a different background*,* […] different religious confessions*,* perhaps also a different understanding of (…) illness and also treatment*,* yes? They might not be as familiar with my concept of orthodox medicine and as convinced of it as I might be. […] I think that a doctor should be open to listen to this*,* to possibly get involved and to respond to the patients’ concerns that they raise.”*

(Interview_03, Paragraph 6)

From students’ point of view, it is not empathetic to judge or pigeonhole patients or even to make fun of or jokes about them.

#### Taking patients’ complaints and needs seriously

Being empathetic as a doctor also means taking patients’ complaints, worries and fears seriously and not dismissing them, even if they do not seem so important or relevant. Interviewee PJ_02 describes how he behaved empathically in a situation with a patient:

*“That you listen first […] and recognize that the person is afraid. Or maybe they are a bit worried before an examination*,* that you take it seriously and don’t dismiss it as ‘Oh*,* don’t be like that.’ or something. And that you then encourage them a little bit*,* ‘It’s not so bad*,* it doesn’t hurt so much.’”*.

(Interview PJ_02, paragraph 14)

#### Holistic view on patients

Medical students’ understanding of clinical empathy includes a holistic view on patients. Interviewees emphasized that it is important not only to consider physical aspects in the patients’ treatment (e.g., laboratory values). To be empathetic one needs to consider patients’ emotional and psychological state in addition to the physical condition.

*“Well*,* I think you have to have […] the ability to say/ understand what/ what the patient’s actual problem is. Not only to see the illness*,* but to understand what it does to the patient and why it is a problem for the patient*,* and you can only do that if you empathize with the patient*,* so to speak*,* and so that they can understand their actual problem. If you don’t do that*,* you often miss the actual suffering of the patient with your treatment.”*

(Interview PJ_07, paragraph 2)

Another way medical students expressed a holistic view on patients was by saying that being an empathic physician means to see every patient as an individual and not to treat patients like “numbers” (Interview PJ_11, paragraph 22) or “cases” that need to be dealt with (Interview_01, paragraph 34).

#### Closeness and familiarity between physician and patient

Friendly, relaxed, and informal communication with patients was also regarded as a component of empathetic interaction. This includes being nice to patients, to smile, to talk in a friendly and warm manner, but also to have fun together or to have conversations about non disease-specific topics like hobbies or the last football game. Moreover, showing one’s own emotions such as joy about progress or sadness is considered empathetic.

*“So*,* somehow a certain kindness*,* which I would also find helpful*,* is*,* I think*,* a certain/ well*,* just being nice. Well*,* there’s a difference between being somehow respectful and*,* let’s say*,* being honest*,* but also being nice. I think that is/ somehow creates closeness to the patient.*

(Interview_05, paragraph 6)

The students believed that creating a spatial proximity between patients and therapists is empathic. This includes for example to move closer to patients while talking with them, to sit down in a conversation, or to meet patients at eye level. Interviewees also experienced a discreet touch on the shoulder or hand of the patient or a hug as empathic. However, students stated that body contact is not appropriate for all patients but must be decided individually.

*“Then I think it’s very important to get down to the level of the patient. You see it quite often*,* I see it here at the university every day. The doctors stand at the bedside*,* preferably at the foot of the bed*,* comfortably far away*,* and if you’re lucky*,* there are also students […] Then a huge white wall is built up and then the question comes*,* ‘Yes*,* do you still have questions?’ No*,* I think that you should also be statically at the patient’s level*,* so to speak*,* concerning your position. In other words*,* if the patient is lying in bed*,* at least take a chair and sit next to them*,* so that you also signal non-verbally*,* ‘I’m with you now*,* I’m not standing over you.’”* (Interview_03, paragraph 86).

Moreover, students thought it empathic if physicians conduct conversations with patients in a quiet and single room and not, for example, in the corridor of the emergency room. Choosing or creating a quiet environment can create more closeness to patients.

#### Treating patients respectfully

According to the medical students, another aspect of clinical empathy is treating patients in a respectful manner. Thus, they thought that it is emphatic to welcome patients at the beginning of a conversation or an examination, also to introduce oneself by name and function or to shake hands, or to tell patients that they will now be taken care of. Interviewee 03 describes empathic communication with patients from his point of view as follows:

*I would separate that […] into verbal and nonverbal. And start with the nonverbal […] It starts with not having patient conversations in the hallway, I think. Not with an open door to the consultation room. If there is someone else in the room it would either be another fellow patient and/or if wanted a relative but no relatives of other fellow patients. […] And then I find it very important to be on the same level as the patient […], to signal nonverbally “I’m with you, I’m not standing above you.” You introduce yourself with your name and position […] You shake hands, well, all kinds of things you get taught, at least I did. [laughs] Yes, well.*

(Interview_03, paragraph 83–86)

An empathic physician addresses patients by their name and talks directly and respectful to them. Students experienced a doctors’ behaviour as less empathic, if doctors talked during a medical round or a lecture in third person about patients and/or used a disrespectful language [e.g. “Yes, here lies our vessel wreck.” (Interview PJ_01, paragraph 76), “Yes, and here you see grotesquely swollen legs.” (Interview_05, paragraph 38)].

It was considered empathic to talk in a language comprehensible to medical laypersons, meaning to describe medical terms comprehensible or to use simple, figurative, and everyday language. Moreover, students thought that empathic behaviour involves to take time to inform patients about their medical situation, upcoming medical examinations, procedures and time frame of examinations, treatments, and diagnostics. A student described the behaviour of a physician that he perceived as very empathic:

*“There was a doctor […] who always explained in detail to the patients ‘What are we going to do now?’*,* introduced herself*,* introduced us all*,* said: ‘Okay*,* we would like to talk to you.’*,* and of course you ask the patient beforehand ‘Is it okay?’ […] And she always explained in detail to the patients*,* so to speak*,* ‘What are we going to do now?’ and it was just such a very/ yes*,* it was just such a very respectful behaviour. It was actually the kind of behaviour where you would think ‘OK*,* that should be normal.’”*.

(Interview_05, paragraph 40)

An honest and authentic approach to patients is also considered empathetic. From the students’ point of view, this means, for example, telling patients when treatment options are limited. Fake kindness is perceived as inauthentic and not empathic. An empathic physician also respects patients’ privacy. This means, for example, to conduct examinations with the room’s door closed, not to talk to patients in the presence of (uninvited) others or not to deliver information in inadequate settings.

### Overarching theme 2: Empathy as an interpersonal, intangible construct

Interviewees understood empathy in the clinical context not only as specific behaviours and attitudes. From their point of view, empathy is also something interpersonal that is not really tangible. Medical students’ expressions for this are, for example, the establishment of an “interpersonal level” (Interview PJ_01, paragraph 32) or a “click moment” between two people, being on the “same emotional level” (both Interview PJ_08, paragraph 18), the transport of “interpersonal vibrations” (Interview PJ_01, paragraph 128) or also the communication of “human warmth” (Interview PJ_01, paragraph 44). This “construct” that can arise between two people enables mutual understanding (Interview PJ_08, paragraph 2). Interviewee PJ_02 talked about her GP and what might make her empathetic:

*“Perhaps the wavelength*,* which is difficult to explain*,* also played a role*,* that you simply feel better off with one doctor than with another*,* even though the other one might make just as much effort*,* is friendly*,* smiles*,* takes his time*,* you somehow feel better off with the other one. So*,* chemistry*,* wavelength*,* I can’t describe it at all*,* but that’s the way it is with my general practitioner*,* for example*,* I’ve felt in good hands there for years*,* even though she may not have unlimited time for me*,* but she seems very understanding and also deals with the problems you describe and doesn’t dismiss it lighthearted*,* but also takes something serious if you communicate it in such a way that it’s important to you to be taken seriously.”*

(Interview PJ_02, paragraph 4)

### Overarching theme 3: taking time for patients

Giving time was seen as a superordinate aspect or maybe a prerequisite of an empathetic approach. However, this aspect was almost always mentioned in connection with other empathy components. Interviewee_12 described, for example, that an empathic doctor behaves appreciatively and respectfully towards patients and takes their problems seriously:

*“Respectfully in any case. As a person*,* as an individual*,* not as a patient with whom I am supposed to make money*,* but really as a human being*,* that’s what I think is important. And*,* as I said*,* not as it should be in some GP practices*,* like working through the patients on an assembly line*,* but really taking time for their complaints.”*

(Interview_12, paragraph 8)

According to the students it is empathetic to take your time, even outside of ward rounds or even when one does not actually have time, to listen to patients, to ask questions, or to explain further therapeutic and diagnostic procedures. It is also empathic to take your time for patients to have space to ask their own questions or to be able to report their view of the disease.

## Discussion

By talking with medical students about empathy we found empathy to be understood as perceiving and understanding patients’ needs and acting accordingly. Showing interests, impartiality and openness towards the patients are subordinate aspects of this definition as well as the need to take patients seriously, treating them with respect, having a holistic view on the patients and generate some kind of closeness with the patients. Empathy was viewed as a relational construct and empathic behaviour seems as being influenced by having an (in)adequate amount of time in clinical practice. This was shown by the fact that the students experienced it as empathetic to take time for patients and their needs.

The students’ definition of empathy reflects Mercer & Reynolds’ [19] definition in some fundamental aspects. Both definitions include cognitive, emotional and behavioural aspects by defining empathy as the ability to perceive, understand, and act upon patients’ emotions. This finding is rather unsurprising as Mercer & Reynolds [19] definition of empathy is one pillar of the development of educational interventions to train medical students in empathy. Kötter et al.’s [43] findings from another German university also support students definition of empathy to resemble Mercer & Reynolds’ [19]. Therefore we can conclude that empathy can be taught in the medical curriculum and students are able to integrate the notion of empathy (see also [44]). Interviewees definition of empathy seem to go beyond a narrow definition of empathy and include aspects of shared-decision making [45] and information giving [46], reproducing the focus on patient-centeredness in the medical curriculum and mixing it under the label of empathy. Schwartz et al. [21] found similar results in physicians.

Our findings are in line with findings from other studies on definitions of empathy by medical students in Mauritius [47], physicians from diverse specialities [18, 21], GPs [17], oncologists [48] and other diverse groups [including oncology patients and non-medical students [12]]. For example, similar to our interviewees, GPs [17] and medical students [47] underline the importance of having sufficient time to be a requisite for empathy exertion. Schwartz et al.’s [21] finding on the importance of making time for patients also correspond to what our interviewees emphasize. Oncologists [48], medical students [47] and physicians [18] also recur to concepts similar to those known from the literature (e.g [19, 49]) as our interviewees did. We did not aim to compare medical students’ definition of empathy with the concepts of the general public, but our results seem to be in line with Hall et al.’s [12] results that show a strong relationship orientation in physicians’ and cancer patients’ concept of empathy in opposition to other groups.

Interviewed students’ definition of empathy included interest in patients, impartiality and openness to their emotions, life situations and needs. Main et al. [50] supports this view by claiming the importance of not only perceiving and labelling other persons’ feelings, but maintaining curiosity towards the reasons for those feelings. Just (possibly inappropriately) labelling feelings without exploring reasons or context respectively may lead to a lower patient satisfaction with medical students’ or physicians’ empathy. Archer et al. [47] also report that medical students see a non-judgemental approach to the patients as an element of empathy.

Despite the fact that empathy is often defined on an intrapersonal level [50], it is an interpersonal process by nature [50, 51]. The relational nature of empathy is often neglected in research settings trying to assess (not only) medical students empathy [50]. Our interlocutors also stressed the importance of the interrelational nature of empathy, talking about finding an interpersonal level or being on the same wavelength with patients which is in line with, for example, findings from a study with Mauritian medical students emphasising the relational nature of empathy [47]. This also explains why in other studies demanding, unresponsive or irritating behaviour is often mentioned as an impeding factor for the exercise of empathy (e.g [7, 52]).

The students’ statements indicate a close connection between empathic behaviour and “having enough time for patients”. (Sufficient) time seems to be an important prerequisite of empathy. Morse et al. [53] found in their study on empathy in lung cancer communication that physicians often miss opportunities for empathy and propose different potential reasons why (subjectively perceived) time constraints might be among the reasons: Busy physicians might assume that empathic communication prolong consultations (which is probably not true [54]) or are distracted by other tasks like diagnosing in limited time [53]. Either way, subjectively perceived time constraints seem to hinder empathic behaviour. Archer et al.’s [47] recent and other researchers’ (e.g [26, 27]) findings support the importance of time for the expression and implementation of empathy.

### Strengths and limitations

There are some studies concerning barriers and facilitators of empathy development during medical studies ( e.g [7, 55]). and physicians definitions of empathy (e.g [18]), but to our knowledge this is the first to study German medical students’ views on and definition of empathy except the study of Kötter et al. [43] asking ten students (members of a selection panel) about their definition of empathy. Therefore our study fills this gap in knowledge revealed by Costa-Drolon et al.’s review [24].

We could only interview students from one medical faculty in Germany. However, we see some consistence with results from other studies (e.g [16, 24, 43]) . As study participation was voluntary we cannot rule out that students highly motivated or more knowledgeable concerning the topic of empathy dominantly self-selected into our study. Either way, we tried to maximize the depth and breadth of participants account by selecting participants to have varied genders and amounts of experience. Today, the data collection was about 8 years ago. As the medical degree program in Hamburg has not changed during this time and because there is no reason to assume that the social or especially the clinical definition of empathy in Germany has changed, we assume that our results are still current and valid.

## Conclusion

Although it is often stated that the various existing definitions of empathy are abstract or far from practice [10, 56, 57] German medical students seem to have a definition of empathy resembling definitions known from the literature [13, 19] and used in education. This suggests that we are managing to teach relatable empathy concepts in medical education in Germany.

Our study underlines the importance of sufficient time to foster empathy in medical care. The problem of not having enough time in medical care resulting in stress [58], burnout [59] and adverse effects on the physician-patient-relationship [21] and patient outcomes [60] is still unresolved. Interventions to foster empathy might focus on dealing with time pressure and stress reduction exercises [61, 62].

Further research is needed to compare concepts of empathy of medical students from different countries and cultural backgrounds. It would also be interesting to investigate how concepts of empathy change over the course of study and affect perceptions of empathy in third party (e.g. patients’) assessments. The relevance of time and the relational quality of empathy should be further explored [47]. Another promising approach would be to compare medical students’, physicians’ and patients’ concepts of empathy (like [12] already did) and bring those together to build an integrated model of medical empathy to base future teaching concerning medical students’, physicians’ and nurses’ behaviour and communication and research on.

## Data Availability

The datasets generated and analysed during the current study are not publicly available due to the assurance to participants that the full raw data would not be shared publicly, and that all attempts would be made to maintain confidentiality, but are available from the corresponding author on reasonable request.

## Declarations

### Authors’ informations

NJP is a female trained psychologist and senior scientist with comprehensive experience in conducting focus groups and interviews as well as qualitative data analysis. AS holds a bachelor degree in physiotherapy and a master degree in health sciences, she is a female researcher with some experience in interviewing and qualitative data analysis. MS is a male full professor of Medicine and board certified in General Medicine.

## Abbreviations

GP: General practitioner
